# From dogs to bats: Concerns regarding vampire bat-borne rabies in Brazil

**DOI:** 10.1371/journal.pntd.0010160

**Published:** 2022-03-03

**Authors:** Marco Aurélio Horta, Leandro Augusto Ledesma, Wlamir Correa Moura, Elba Regina Sampaio Lemos

**Affiliations:** 1 BSL-3 Facility, Oswaldo Cruz Institute, Oswaldo Cruz Foundation, Rio de Janeiro, Brazil; 2 Instituto Nacional de Infectologia Evandro Chagas, Oswaldo Cruz Foundation, Rio de Janeiro, Brazil; 3 Instituto Nacional de Controle de Qualidade em Saúde, Oswaldo Cruz Foundation, Rio de Janeiro, Brazil; 4 Laboratory of Hantavirus and Rickettsiosis, Oswaldo Cruz Institute, Oswaldo Cruz Foundation, Rio de Janeiro, Brazil; US Department of Agriculture, UNITED STATES

## Background

Human rabies is a neglected reemerging disease that has a major impact on public health in poor communities and low- and middle-income countries [1]. With a lethality of almost 100% worldwide, rabies has an incidence of approximately 59,000 cases per year in 150 countries [2]. Rabies is transmitted by exposure to the saliva of infected animals, and it is 100% vaccine preventable [3]. Every year, more than 15 million people worldwide receive pre-exposure prophylaxis and 29 million receive a post-bite vaccination, which is estimated to prevent thousands of rabies deaths annually [2]. With a worldwide distribution of cases of the disease, the largest number occur in Asia and Africa, which together account for 95% of all human cases, with 99% acquired from the bite of an infected dog [4]. Globally, canine rabies causes approximately 59,000 human deaths annually [5].

Vaccination is the main effective strategy for preventing fatal viral diseases. Considering the dog-mediated urban cycle in areas of high endemicity, the only way to stop the transmission of rabies is to vaccinate at least 80% of the canine population [6]. Vaccinating the highest number of dogs in a region creates herd immunity, slowing the progression of rabies infections among dogs, and reducing the chances of transmission. In Americas, approximately 100 million dogs are vaccinated annually. Furthermore, the World Health Organization has implemented the global strategic plan to end human deaths from dog-mediated rabies by 2030, focusing on the regional goal of eliminating rabies by strengthening programs to improve their surveillance and vaccination coverage for dogs in the areas most at risk [6]. Considering the growing importance of the sylvatic cycle where the RABV lineages are maintained in different independent epidemiologic cycles in the Americas by wild animals, particularly chiropterans [7], in this article, we will discuss the change in the epidemiological profile of rabies in Brazil, from dogs to bats as the main reservoirs of the disease today and how this impacts the surveillance and disease control actions.

## Bats as the main rabies transmitters in Brazil in the recent years

Brazil has experienced a sharp decline in the number of rabies cases in recent decades. In 1990, 72 cases were reported, and, in 2020, the Brazilian Ministry of Health reported only 1 case by bat transmission. There was also a change in the form of transmission of the rabies virus. In 1990, 50 cases of rabies were transmitted through a dog bite, 2 cases by a feline bite, and 11 cases by a bat bite. In 2017, among the 6 reported cases, none was related to a dog bite, 5 were attributed to a bat bite, and 1 was attributed to a feline bite. In 2018, the 11 reported cases were attributed to bats, and, in 2019, the only reported case was caused by a feline bite infected by the bat antigenic variant AgV-3 [8]. These observations make rabies control more difficult once the measures to control bats populations are not indicated in Brazil. Bat-transmitted rabies must be in the scope of the surveillance health services and once a notification is confirmed, it is necessary to mobilize and manage the resources required for the rapid response, which includes control vaccinations in cases of outbreaks and a widespread public concern. It is important to keep the public informed about the human rabies outbreak and outbreak response.

**Fig 1:**
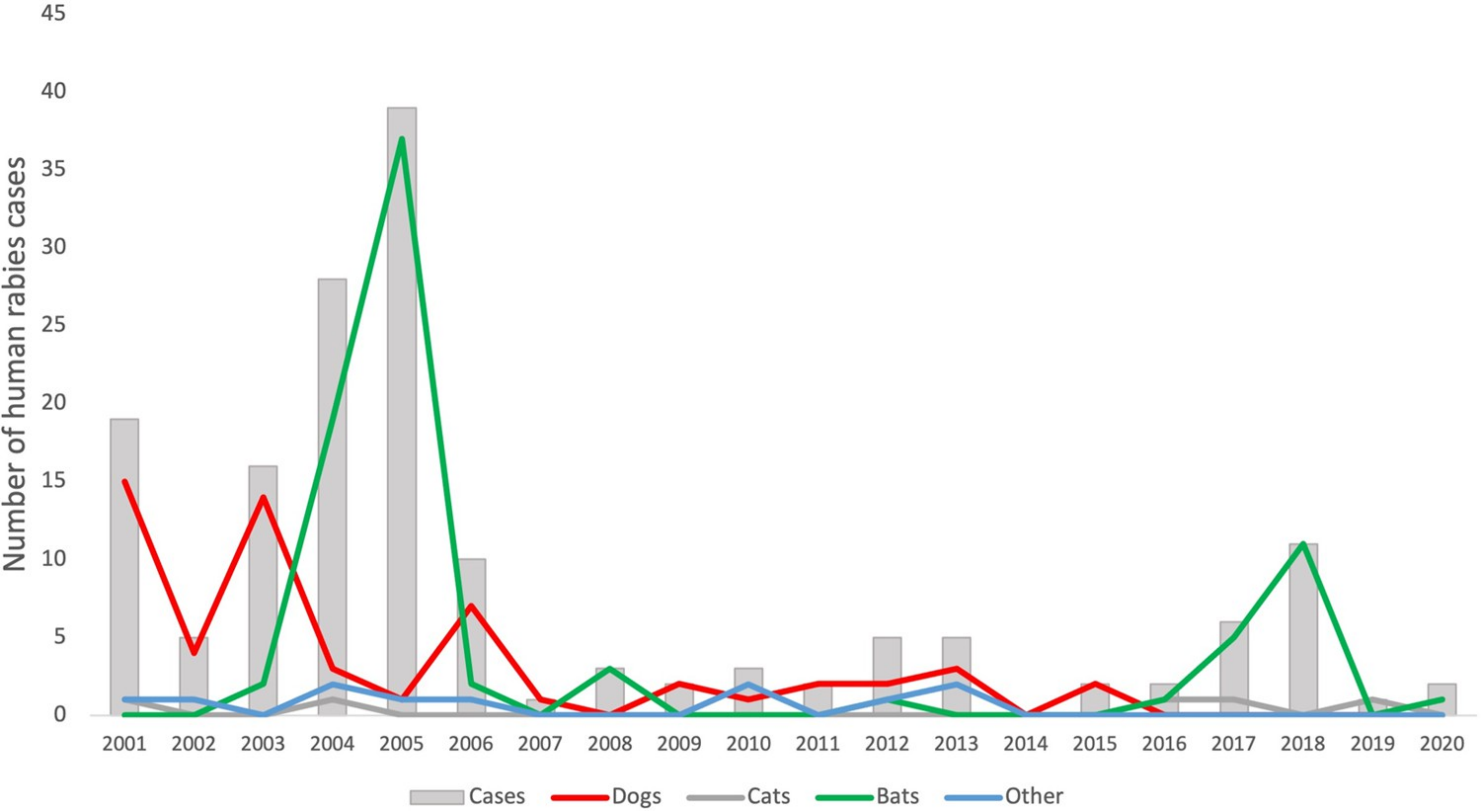
Human rabies cases following exposure to dogs, cats, bats, and other wild carnivore host species in Brazil.

The change in the epidemiological profile of rabies in Brazil places bats as a key point in the current transmission chain (Fig 1). Although several bat species have been confirmed as rabies positive, *Desmondus rotundus* (vampire bat) is mainly responsible for the transmission of the rabies virus to the human population. To confirm the actual role of bats in rabies transmission, in 24.0% (*N =* 150) of human rabies cases confirmed between 2000 and 2017, the most common viral variant was AgV3 (from *D*. *rotundus* bats), also found in 3 transmissions by cats, followed by AgV2 (*Canis lupus*), AgVnC (*Callithrix jacchus*), and AgV1 (*C*. *lupus*) [9]. These hematophagous bats exclusively feed on blood and are responsible for the transmission of various emerging and zoonotic pathogens in addition to rabies [10]. At night, these sanguivorous mammals prey on other animals, such as horses, cattle, dogs, and even humans. Exposure to blood-eating bats usually occurs in regions with alterations in their habitat when the population of natural prey decreases. The availability of food and roosts for *D*. *rotundus* may be directly influenced by land use patterns, and it represents a type of transformation of the environment that may invoke risks to human health [11,12,13]. In addition, small villages, such as those in rural Amazonia settlements, with vulnerable dwellings associated with difficult access to healthcare facilities may reduce the chance of post-exposure prophylaxis and are also more likely to experience vampire bat predation [14]. Power failure and <17 years of age were risk factors for a human rabies outbreak in 2009 after an attack by vampire bats [15], in places where bat bites were considered normal.

As part of the land-use changes, although canine rabies is successfully controlled in Brazil, bovine conditions are still endemic in many regions of the country. Rabies is an important zoonotic viral disease in livestock in Brazil, with an estimated annual loss of 850,000 heads, equivalent to 17 million US dollars [16], and is determined mostly by the presence of the vampire bat *D*. *rotundus*. Recently, the efforts of the National Program for Rabies Control in Herbivores in reducing the disease prevalence in the domestic herbivore population have been successful [17]. Bovine notifications dropped from 555 to 317 cases from 2017 to 2019 [6], although these data may be biased by a possible underreporting of cases owning to a lack of systematic collection of samples for laboratory analyses. In the past decades, the movement of the animals during the dry season in the Brazilian Northeastern region, where it is common to transport cattle to a different region to save them from drought and starvation, accounted for the occurrence of human outbreaks since vampire bats were forced to find another source of food, including human beings [18]. Because *D*. *rotundus* is becoming relatively common in regions with large herds of domestic cattle, which may result in an increase in survival rates and population density, especially in areas where appropriate roosts are available, the bovine serves as sentinels for those regions that have the rabies virus circulating [11]. This intimate relationship between the density of positive cases of rabies and expansion of cattle herds in Amazonia has been reported in recent literature [11].

## Challenges for Brazilian health surveillance

Vaccination campaigns for domestic animals in Brazil have caused a sharp reduction in cases of human rabies transmitted by dogs, and since 2015, no cases by canine variant have been reported [6]. However, some municipalities have postponed or suspended animal rabies vaccination campaigns because of the lack of transfer of vaccines sent by the Brazilian Ministry of Health to state governments. Other municipalities have decided to postpone the 2020 vaccination campaign against rabies for dogs and cats as a preventive measure against COVID-19 in order to keep social and physical distancing and avoid the risk of overcrowding. Usually, these actions take place annually between August and September. The campaigns will possibly be rescheduled depending on the epidemiological scenario of the coronavirus disease. This change in the epidemiological profile of rabies in Brazil was also accompanied by a change in the administered doses of anti-rabies serum, from 11,000 doses in 2001 to 112,000 in 2010 and from 91,000 doses in 2011 to 11,000 in 2019. However, the amount of administered doses of rabies vaccine has been decreasing in recent years. From 2015 to 2020, the number of doses was 1 million, 583,000, 443,000, 335,000, 260,000, and 127,000, respectively [6]. The risk of vaccine and anti-rabies serum stock shortage for rabies control is a threat to the successful programs for the control and elimination of dog-maintained rabies in Brazil considering the pre- and post-exposure prophylaxis [19]. Not only availability, but also high rates of inadequate post-exposure rabies procedures in some Brazilian states should be a concern for health authorities [20].

In 2019, a 58-year-old woman living in a rural area in the municipality of Gravatal, State of Santa Catarina, southern Brazil, died of rabies after a cat bite. However, Santa Catarina has not registered a case of rabies in humans since 1981. The last cases of rabies in dogs and cats in Santa Catarina were registered in 2006 and 2016. These cases are warning signals for health authorities because they occurred in areas where rabies cases have not been reported for many years. In 2020, the Rio de Janeiro State Health Department reported a new death due to rabies in the rural area of Angra dos Reis city, which was the first case of death from rabies in the state since 2006 that was transmitted by a bat [8]. These facts may or may not be associated with changes in natural landscapes or even the effects of climate change.

Climate conditions may influence the dynamics of bat populations and, consequently, rabies cases. Several studies on the spatiotemporal distribution of rabies virus in bats and other mammal species have shown the influence of factors, such as temperature, rainfall, and the El Niño Southern Oscillation, on the occurrence of rabies outbreaks in certain areas, seasons, and throughout the year [17,21]. In a climate change scenario, bats species might expand its range in response to increasing temperatures [22] and the mechanisms behind bat range shifts in response to climate are likely to include dependence on water, bat physiology, phenology, and roost quality [23,24]. As part of a multifactorial system that includes deforestation, land use, exchange of natural ecosystems for pastures where intensive livestock production keeps a large number of animals [25], and changes in local and regional climate due to global warming could alter areas of bat species and lead to the occurrence of cases of human rabies in places without previous notification of the disease. Modeling future scenarios for sanguivorous bats in the US border with Mexico, authors have shown regions in southern Texas that may become suitable for the species occurrence by year 2070. However, because of the degree of uncertainty, the climate models are not unanimous in conclusions for future scenarios [26]. More studies are necessary to model rabies distribution under different levels of global warming and climate change scenarios. Human rabies transmitted by bats has become significant in Brazil in the last few decades. Recent data show that the rabies virus can spread to areas without a prior record of the disease. The number of dog-mediated human rabies in Brazil declined in the last decades, from 15 in 2001 to 0 in 2021. This decline can be attributed to successful pet vaccination, public health surveillance, and availability of post-exposure prophylaxis for rabies. However, factors like the habitat fragmentation, range shifts by bats that have coincided with temperature increases, and the growing availability of livestock as a food source for vampire species may have favored transmission of rabies by bats in recent years in Brazil.
